# Pathophysiology of Diverticular Disease: From Diverticula Formation to Symptom Generation

**DOI:** 10.3390/ijms23126698

**Published:** 2022-06-15

**Authors:** Maria Raffaella Barbaro, Cesare Cremon, Daniele Fuschi, Giovanni Marasco, Marta Palombo, Vincenzo Stanghellini, Giovanni Barbara

**Affiliations:** 1 Division of Internal Medicine, IRCCS Azienda Ospedaliero-Universitaria di Bologna, 40138 Bologna, Italy; maria.barbaro2@unibo.it (M.R.B.); cesare.cremon@aosp.bo.it (C.C.); daniski91@gmail.com (D.F.); giovanni.marasco4@unibo.it (G.M.); v.stanghellini@unibo.it (V.S.); 2 Department of Medical and Surgical Sciences, University of Bologna, 40138 Bologna, Italy; marta.palombo2@unibo.it

**Keywords:** diverticular disease, SUDD, pathophysiology, genetic factors, environment, diet, microbiota, inflammation, ENS

## Abstract

Diverticular disease is a common clinical problem, particularly in industrialized countries. In most cases, colonic diverticula remain asymptomatic throughout life and sometimes are found incidentally during colonic imaging in colorectal cancer screening programs in otherwise healthy subjects. Nonetheless, roughly 25% of patients bearing colonic diverticula develop clinical manifestations. Abdominal symptoms associated with diverticula in the absence of inflammation or complications are termed symptomatic uncomplicated diverticular disease (SUDD). The pathophysiology of diverticular disease as well as the mechanisms involved in the shift from an asymptomatic condition to a symptomatic one is still poorly understood. It is accepted that both genetic factors and environment, as well as intestinal microenvironment alterations, have a role in diverticula development and in the different phenotypic expressions of diverticular disease. In the present review, we will summarize the up-to-date knowledge on the pathophysiology of diverticula and their different clinical setting, including diverticulosis and SUDD.

## 1. Introduction

Diverticular disease (DD) is an extremely common condition in industrialized areas, with a high incidence in Western countries [1,2,3]. The prevalence of colonic diverticula is about 5% before the age of 40, while it increases to 50% around 60 years, and exceeds 71% after 80 years [4].

The term DD is often used to describe asymptomatic diverticulosis, which is usually detected incidentally in patients undergoing colonoscopy or abdominal imaging and proportionally increases with age [5]. Although in most cases diverticulosis remains asymptomatic [5], approximately one-quarter of patients may develop clinical manifestations. In terms of severity, DD is classified into symptomatic uncomplicated diverticular disease (SUDD) and symptomatic complicated disease such as acute diverticulitis (with or without complication) or diverticular hemorrhage. SUDD is a subtype of DD characterized by irritable bowel syndrome (IBS)-like symptoms, including abdominal pain, that can be attributed to diverticula, without macroscopic evidence of overt inflammation or other complications [6,7,8]. Diverticulitis is the most common complication of diverticulosis, occurring in approximately 1% of patients over 11 years [9]. The pathophysiology of diverticulitis appears to be related to obstruction of the diverticulum by fecal material, which causes inflammation of the mucosa resulting in congestion, mucosal trauma, and ischemia [10]. Complicated disease, occurring in about 12% of patients with diverticulitis, is generally characterized by the formation of perforation, abscesses, obstruction and/or fistulas [11] and may require surgery [12,13].

For many years, DD has been considered exclusively an age-related condition. New evidence demonstrated that genetic, environmental factors, and changes in the intestinal microenvironment, including microbiota imbalance, mucosal inflammation, enteric nervous system (ENS), and neuro-immune alterations, play a pivotal role in the pathophysiology of diverticulosis and SUDD. In addition, changes in the architecture of the colon wall, including enhanced collagen crosslinking and reduced elasticity, are also involved in the pathophysiology of DD [10].

In this review, we will discuss the evidence on pathophysiological mechanisms underlying DD, principally focusing on the factors involved in diverticula development and symptom generation in diverticulosis and SUDD.

## 2. Genetic Factors

Historically, environmental risk factors were largely investigated, emerging as fundamental in DD evolution. Despite this, numerous data suggest a role for genetic factors among the causes predisposing to DD.

One evidence relates to the high prevalence of diverticulosis in Western countries (>50% of people aged over 60), together with the different distribution of diverticula through the right and left side of the colon in Western and Eastern populations, respectively, that unlikely relate to differences in environmental factors only [14]. In support of a key role of genetic susceptibility to DD are studies of migration, which demonstrated that the incidence of DD did not change as a result of a shifting lifestyle [11]. The Japanese population living in Hawaii shows the canonical predominance of right-sided diverticula, although adopting a Westernized diet [15]. A study carried out on the London Bangladeshi population displayed a very low prevalence of colonic diverticulosis in this ethnic group (2.7%), compared to Caucasian (36%), Indian/Pakistani (10%), Oriental (34.9%), and Black (24.4%) individuals living in the same region [16]. A similar observation, corroborating the contribution of genetic factors to DD pathophysiology, emerges by comparing the incidence of diverticulosis in Turkish migrants in the Netherlands (7.5%) with the native Dutch population (50%) [17]. Furthermore, monogenic disorders of connective tissue, as well as Coffin-Lowry syndrome, Marfan syndrome, Williams-Beuren syndrome, Ehlers–Danlos syndrome, and Autosomal Dominant Polycystic Kidney Disease are characterized by colon diverticula at an early age, suggesting the involvement of the same genetic factors in diverticula formation too [18,19,20]. Population-based familial aggregation studies allowed to estimate the heritability of DD at around 45% and the contribution of the environmental factors at55% [21,22]. These studies identified a higher risk of hospitalization for DD in individuals with siblings affected by the same disease than in the average population. Analogously, the probability to develop DD increases in monozygotic twins compared to dizygotic [21,22].

Moreover, case-control studies strengthen the idea of a genetic background predisposing to DD, highlighting an association between the disease and single nucleotide polymorphisms (SNPs) (Table 1). This approach allows identifying the genes *COL3A1*, *TNFSF15*, *RPRM*, and *LAMB4* encoding respectively for: type III collagen, a cytokine of the TNF family, the protein Reprimo involved in DNA repair and cell cycle regulation, and a constituent of the extracellular matrix named laminin subunit β4 [23,24,25,26]. Recently, the SNP rs4898 of gene *TMP1* was associated with an increased risk of diverticulosis [27]. *TMP1* encodes for a tissue inhibitor of metalloproteinases, which regulates collagen extracellular matrix degradation [28]. This observation both highlights the presence of a genetic predisposition to DD and enhances the idea that collagen cross-linking alterations are involved in the pathogenesis of this disease. Additionally, genome-wide association studies (GWAS) provided the most important data on genetic susceptibility in DD (Table 1). The first GWAS in the Icelandic and Danish populations identified different loci linked to the disease and located in the intronic region of *ARHGAP15*, *COLQ*, and *FAM155* genes [25]. Specifically, the *ARHGAP15* and *COLQ* variants associate with uncomplicated DD, whereas *FAM155* mutation is linked to diverticulitis, but not with diverticulosis [25,29]. The *ARHGAP15* gene encodes the Rho GTPase activating protein 15, linked with phagocyte function and inflammation [30]. Differently, *COLQ* encodes the collagen-like tail subunit of acetylcholinesterase involved in postsynaptic differentiation at the neuromuscular junction. Mutations of the gene can compromise acetylcholinesterase availability and extend nerve to muscle signaling, resulting in alteration of muscle functionality [31] The FAM155 family consists of two members in humans, FAM155A and FAM155B, which are likely to be involved in the sodium leak channel (NALCN) function and stabilization [32]. The NALCN multi-protein complex is a sodium-selective and voltage-modulated ion channel essential for setting membrane excitability [33]. It is implicated in several physiological processes including circadian and respiratory rhythms [34,35]. The homolog of FAM155 in *C. elegans* (NLF-1) has been demonstrated to be essential for the membrane localization of the NACLN complex [36]. Additionally, GWAS, involving 28,000 patients with DD and sampled in UK Biobank, identified 42 risk loci, which are related to genes associated with the extracellular matrix, membrane transport, immunity, cell adhesion and structures controlling intestinal motility. In addition, this study confirmed the previously identified genes *ARGAP15*, *COLQ*, and *FAM155A* [37]. Finally, the more extended GWAS performed on 31,964 cases and 419,135 controls and replicated in 3893 cases and 2829 controls, allows identifying 48 loci associated with DD, of which 12 new [38]. This study shows that DD is a disorder of impaired intestinal neuromuscular function, mesenteric vascular, smooth muscle, and connective fiber support, while epithelial alteration should be associated with an increased risk of diverticulitis [38]. A GWAS study was conducted on the Korean population to identify genetic factors involved in DD. This study was carried out on 893 patients with right-sided diverticulosis or bilateral, and 1075 controls, and replicated on 346 patients and 305 controls. Although this study has a statistical limitation, as the Bonferroni correction deleted the statistical significance, it highlighted 9 SNPs located in 3 new candidate genes: *WNT4*, *RHOU,* and *OAS1/3* [39]. Both the *WNT4* and the *RHOU* genes encode for members of the WNT signaling pathway. This protein family is involved in the homeostasis of the intestinal epithelium [40] and the development of the gut [41]. Moreover, the WNT signaling is associated with intestinal glial and neuronal differentiation in rats, together with anti-inflammatory activity in the enteric nervous system (ENS) [42]. Differently, the members of the OAS family are protein induced by interferon and linked to antiviral and apoptotic responses [43], strengthening the idea of chronic low-grade inflammation in DD pathophysiology [39].

## 3. Environmental Factors

### 3.1. Dietary Fibers

The geographic distribution of the DD and its correlation with a Western diet has suggested a role for dietary factors in the pathogenesis of the disease. Burkitt and Painter first proposed the DD as a disorder that could be averted with dietary change. Their theory is based on the assumption that fibers affect transit time and stool weights. Specifically, they analyzed more than 1200 individuals, from the UK or rural Uganda, and inferred that the low-fiber Western diet produces smaller stool volumes and longer transit time. In turn, these effects could predispose to increasing intraluminal pressure, hence diverticular herniation [44]. A prospective study involving 43,881 men demonstrated that a high fiber diet, and precisely soluble fibers, decrease the risk of DD [45]. A study, performed on a large UK cohort of 47,033 subjects, evaluated the association between a vegetarian diet and dietary fiber assumption and the risk to develop DD [46]. The results demonstrated that after a mean follow-up of 11.6 years, vegetarians had a reduction of 30% of the risk to develop DD compared to subjects consuming meat. In addition, individuals consuming more than 25 g of fibers/day had a decrease in the hospitalization risk by about 40% compared to subjects consuming less than 14 g/day [46]. Taken together these data suggest a protective role of fiber consumption against the development of DD.

However, the association between fiber intake and DD is still controversial. Peery and colleagues performed a cross-sectional study of 2104 subjects, demonstrating that a high fiber diet was associated with a higher prevalence of diverticulosis [47]. A cross-sectional study involving 539 patients with diverticulosis and 1569 controls, demonstrated the lack of association between constipation associated with a low-fiber diet and the risk of diverticulosis [47]. Specifically, subjects with less than seven bowel movements/week had a reduced risk of diverticulosis compared to subjects with seven bowel movements/week. In addition, hard stools were associated with a reduced risk of diverticulosis, while there was no association between dietary fiber intake and diverticulosis [47].

Recently, a systematic review and meta-analysis of prospective cohort studies assessed the association between dietary fiber intake and the risk of DD. Five studies, including 19,282 cases and 856,829 participants, were eligible for the meta-analysis. The results suggest a 41% risk reduction of DD in people consuming a high fiber diet (30 g per day) compared to subjects with a low fiber diet [48]. It can be suggested that gut microbiota mediates the effect of fibers on the host. Indeed, dietary fibers enhance microbial diversity through the production of SCFAs, which have an impact on the host immune system as well as on the mucosal barrier [49,50,51]. Based on the controversial observations on the role of fibers in DD (Table 2), the American Gastroenterology Association (AGA) guidelines recommend a high-quality diet (high in fiber from fruits, vegetables, whole grains, and legumes and low in red meat and sweet) only in patients with a history of acute diverticulitis to reduce the risk of recurrence [13]. Other studies are necessary to clarify the benefit of fiber supplementation in the management of SUDD.

### 3.2. Red Meat Intake, Alcohol, Smoking, and Lifestyle

DD has been associated with the Western lifestyle due to changes in diet and living habits after the industrial revolution. In fact, in Africa, DD has a prevalence of 4%, probably due to a low-fat diet [54,55,56], while in Asia its prevalence is between 8 and 25% [57].

The abovementioned decrease in fiber intake is complemented by other dietary modifications, including an increase in red meat consumption. Aldoori and co-workers highlighted a significant correlation between red meat consumption and DD development, although no dose-response relationships were found [52]. In contrast, Peery and colleagues did not find any link between red meat intake and diverticulosis [53].

A case-control study involved 2164 subjects, comprising 542 patients with asymptomatic diverticulosis, who underwent colonoscopy. The results highlighted alcohol consumption and smoking as risk factors for diverticulosis. In addition, these two risk factors were associated with right-sided and bilateral diverticula [58].

A large prospective study carried out for 10 years in Japan evaluated 16 candidate risk factors in 3327 asymptomatic subjects undergoing colonoscopy from the general population. Results showed a significant positive correlation between diverticulosis and some lifestyle-related factors including smoking, drinking, and severe weight increase in adulthood [59]. Similarly, a prospective study involving 623 patients demonstrated that obesity increased the risk of diverticulosis in women but not in men [60].

Concerning the development of symptoms, a prospective work with a four-year follow-up period showed a weak and non-significant association between alcohol and smoking and the development of symptomatic DD [61]. In addition, physical activity, principally vigorous exercises, such as running, was associated with decreased risk of symptomatic diverticular disease [62].

## 4. Microenvironment

### 4.1. Microbiota

Accepting the concept that diet and lifestyle are key factors involved in DD pathophysiology, these factors have been stringently linked also with gut microbiota composition. Western dietary patterns, as well as obesity, are associated with a decrease in microbial diversity and a change in microbial composition (Table 3) [63,64,65]. Conversely, high fiber intake promotes microbiota diversity [49,66] and enhances short-chain fatty acids (SCFAs) production by microbial species [50], which contribute to shaping immune function and the intestinal mucosal barrier [51]. Based on this evidence it would be conceivably of great interest to explore the role of microbiota in DD, but unfortunately, only a small number of studies have explored this factor in this condition.

In a recent pilot study, we have characterized the fecal and mucosal microbiota of patients with diverticulosis and SUDD and compared the results with those obtained from healthy controls. Patients with diverticulosis and SUDD showed a decrease of *Clostridium* cluster IV in the feces compared to controls [67]. Interestingly, this bacteria group includes several species with anti-inflammatory function and butyrate-producing capability. In the same study, we evaluated the urinary metabolome and identified six metabolites that can discriminate patients with diverticula from controls with >95% accuracy. Interestingly, the metabolome results are consistent with inflammatory response and microbiota alterations [67].

Tursi et al. identified no differences in the fecal microbial composition of patients with SUDD, diverticulosis, or control subjects, except for *Akkermansia muciniphila* that increase in SUDD and diverticulosis [68]. Interestingly, this bacterial species usually is closely associated with the mucosal layer [69], which is used as a source for the production of acetate and propionate [70]. Therefore, its presence in the feces of SUDD patients suggests both a change in the bacterial amount and an alteration at the mucosal layer. Moreover, the *Akkermansia muciniphila* expansion co-occurred with the increment of several SCFAs [68]. Definitely, *Akkermansia muciniphila* is considered a hallmark of a healthy gut. Another small study detected a depletion of *Bacteroides fragilis*, *Collinsella aerofaciens,* and *Collinsella stercoris* species in fecal samples of patients with DD, compared with controls [71]. Conversely, a study involving 226 subjects with diverticula and 339 subjects without diverticula during a screening colonoscopy, evaluated mucosal microbiota by using 16S-sequencing. They found only weak associations between diverticula and decreased abundance of Proteobacteria and Comamonadaceae, concluding that mucosal adherent bacteria did not play a key role in diverticula development [72]. A recent study evaluated the microbiota profiling in the sigmoid and transverse colon of patients with diverticulosis in comparison with healthy subjects. By using a 16S-23S based bacterial profiling technique, authors showed no difference between subjects with and without diverticula; in addition, the microbiota profiling was similar in the sigmoid and in the transverse region [73].

Concerning the association between SUDD symptoms and microbiota alteration, the analysis of stool samples from patients with SUDD highlighted a positive association between bloating severity score and the relative abundance of *Ruminococcus* and a negative correlation between bloating and *Roseburia* amount. Interestingly, the *Ruminococcus* genus ferments polysaccharides to hydrogen in the gut, while species belonging to the *Roseburia* genus are a producer of butyrate that enhances intestinal motility and reduces hypersensitivity. Finally, the same study showed that pain intensity was significantly associated with *Cyanobacterium* number [74]. In our pilot study, we showed that SUDD patients were characterized by a significant reduction of faecal *Clostridium* cluster IX, *Fusobacterium*, and Lactobacillaceae species, compared with diverticulosis [67]. Concerning mucosal samples of patients with SUDD, we found a lower abundance of *Akkermansia muciniphila* in the biopsy taken close to the diverticula, compared to mucosal biopsy from normal mucosa, far from diverticula [67]. In addition, we found an inverse correlation between the abundance of *Akkermansia* and *Clostridium* cluster IV with macrophages in the diverticular region, suggesting a pro-inflammatory effect, which may underlie symptom generation.

**Table 3:** Main studies assessing gut microbiota in DD.

Authors	Subjects (n)	Samples	Microbial Profiling Method	Outcomes
Kvasnovisky et al., 2018 [74]	SUDD (30)	Stools	16S ribosomal RNA gene sequencing	Positive association between bloating severity score and the relative abundance of *Ruminococcus*, and a negative correlation between bloating and *Roseburia* amount. The intensity of pain was significantly associated with *Cyanobacterium* number.
Barbara et al., 2017 [67]	HC (14) Diverticulosis (16) SUDD (8)	Stools Mucosal biopsies	high taxonomic fingerprint (HTF)-Microbi.Array	↓ *Clostridium* cluster IV DD vs. HC in stool samples ↓ *Clostridium* cluster IX SUDD vs. Diverticulosis in stool samples ↓ *Fusobacterium* spp SUDD vs. Diverticulosis in stool samples ↓ Lactobacillaceae SUDD vs. Diverticulosis in stool samples ↓ *Akkermansia muciniphila* in SUDD in mucosal biopsy close to diverticula vs. mucosal biopsy far from diverticula
Tursi et al., 2016 [68].	HC (16) Diverticulosis (13) SUDD (15)	Stools	RT-PCR	*↑ Akkermansia muciniphila* DD vs. HC
Lopetuso et al., 2017 [71]	HC (8) DD (4) IBS (3) UC (5) CD (10)	Stools	16S ribosomal RNA gene sequencing	↓ *Bacteroides fragilis* DD vs. HC ↓ *Collinsella aerofaciens* DD vs. HC ↓ *Collinsella stercoris* DD vs. HC
Jones et al., 2018 [72]	HC (309) Diverticulosis (226)	Mucosal biopsies	16S ribosomal RNA gene sequencing	↓ Proteobacteria vs. HC ↓ Comamonadaceae vs. HC

HC, healthy controls; SUDD, symptomatic uncomplicated diverticular disease; IBS, irritable bowel syndrome; UC, ulcerative colitis; CD, Crohn’s disease; ↓, decrease; ↑, increase.

### 4.2. Low-Grade Inflammation

Although inflammation is a typical hallmark of acute diverticulitis and its complications, the role of low-grade inflammation or immune activation in diverticular formation or symptom generation in patients with SUDD is still a matter of discussion.

The assessment of IL-10 in the feces of healthy subjects, diverticulosis, and SUDD, revealed an increase in this anti-inflammatory cytokine amount in both groups of patients with diverticula, irrespective of the presence of symptoms, although the statistical significance was not determined [75].

To evaluate mucosal inflammation in different stages of DD, Tursi and colleagues enrolled 10 patients with asymptomatic DD, 10 with SUDD, 10 with acute uncomplicated diverticulitis, and 10 healthy subjects. T cell infiltrate was found in all patients with diverticula and showed an increasing trend according to the severity of the disease [76]. In addition, compared to healthy subjects, T cells were significantly increased in all subgroups of patients with DD. In the same study, neutrophils were found only in acute uncomplicated diverticulitis [76].

We analyzed and quantified mast cells, T cells, and macrophages in patients with diverticulosis and SUDD, compared to healthy subjects. In particular, we evaluated immune cells in the colonic mucosa close to the diverticula and in a region without diverticula. We did not find any difference in mast cell and T cell count among groups, while macrophages were significantly increased in both diverticulosis and SUDD compared to healthy subjects. In addition, we found increased macrophage counts both in the diverticular region and in the distant site [67]. These results suggest that macrophages are associated with the presence of diverticula, more than with symptoms.

In line with our results, a large study involving 255 patients with diverticulosis reported no difference in T cells or mast cells in the colonic mucosa, compared to subjects without diverticula. In addition, this study did not find any difference among patients with or without diverticula in the expression of IL-6, IL-10, and TNF-α [77]. One year later, another population-based colonoscopy study on 254 subjects evaluated C-reactive protein (CRP) and histological markers of inflammation. The results showed no difference between controls and diverticulosis for CRP and no association between diverticulosis and inflammation [78]. A recent study evaluated T lymphocytes and neutrophils in the sigmoid and in the transverse colonic biopsies of patients with diverticulosis in comparison with healthy subjects. No difference emerged for T cells between the two groups, while neutrophils were not found in the analyzed samples [73]. Unfortunately, none of these three latter studies evaluated mucosal macrophages.

Concerning the association between low-grade inflammation and symptoms, a study on 47 patients who underwent surgery for atypical “smoldering” DD (i.e., subjects with chronic symptoms but without overt diverticulitis) showed that 76% of resected specimens showed histological features of acute and chronic inflammation [79]. In support of the idea that inflammation is involved in DD, a study by Tursi et al. reported increased fecal calprotectin in acute uncomplicated diverticulitis and in SUDD compared to healthy subjects and IBS patients. The authors speculated that fecal calprotectin could be used to distinguish SUDD from IBS [80].

Low-grade mucosal inflammation was investigated in a study involving 13 asymptomatic patients and 12 patients with SUDD. No difference emerged in 5HT or CD3 lymphocytes in the colonic mucosa of the two groups of patients, while TNF-α and IL-6 mRNA expression were significantly increased in SUDD patients. In addition, this study demonstrated an increased gene expression of the neurokinin 1 receptor (NK1) in the colonic mucosa of SUDD patients [81], suggesting that the development of symptoms in SUDD patients could be mediated by inflammation.

The release of IL-10 by biopsies was evaluated in patients with diverticulosis, SUDD, SUDD subsequent to previous acute diverticulitis and asymptomatic controls. The results showed that IL-10 release was significantly increased in SUDD patients with previous acute diverticulitis compared to asymptomatic subjects [82] probably as a counter-regulatory signal of increased pro-inflammatory cytokines.

A study by Tursi et al., reported significantly increased levels of TNF-α expression in SUDD compared to healthy subjects and asymptomatic patients. The authors concluded that TNF-α expression is related to the severity of the disease [83].

### 4.3. Enteric Nervous System and Neuro-Immune Interactions

Modifications in the ENS structure emerged as early as 50 years ago when Macbeth and Hawthorn [84] described enlarged and ectopically localized myenteric ganglia in DD. The morphometric analysis found a significant reduction in neuronal density in the myenteric and submucosal plexus of DD patients [85]. Compared to control subjects, patients with DD showed a reduced number of ganglia, neurons [85,86,87], enteric glial cells, and intestinal pacemaker cells (interstitial cells of Cajal) [88,89]. Among possible factors responsible for these changes, a deficiency of glial cell-derived neurotrophic factor (GDNF) and its receptors has been found in the muscularis propria of patients with DD compared with controls [90]. In support of this hypothesis, Barrenschee showed decreased mRNA expression of GDNF and its corresponding receptors GDNF family receptor alpha 1 (GFRα1) and Rearranged during transfection (RET) in the tunica muscularis of symptomatic and asymptomatic patients with diverticula [91,92]. These findings corroborate the idea that an alteration in the GDNF system is a key factor in the neuropathy underlying DD [91]. We evaluated nerve fiber density in the colonic mucosa of asymptomatic and symptomatic patients with DD, compared to healthy subjects, in a region close to a diverticulum (diverticular region) and in a site far from the diverticula. Nerve fibers, assessed as NSE positive fibers, were increased in patients with diverticula, irrespective of the presence of symptoms, compared to healthy subjects only in the diverticular region [93], which is in line with previous data [94].

Growing evidence suggests that modifications in the ENS observed in diverticulosis may participate in DD pathophysiology and progression to symptoms [95]. In particular, alterations in the enteric neurotransmitters have been assessed as the possible cause of symptom development. Neurotransmitters with both excitatory (e.g., acetylcholine, substance P) and inhibitory (e.g., nitric oxide, vasoactive intestinal polypeptide) functions have been described to be involved in DD [96,97,98,99]. Data from Simpson et al. support this evidence, since they found in the mucosa of patients with SUDD a 10-fold up-regulation of pain-mediating neuropeptides, such as galanin, neuropeptide K and substance P compared to controls [94]. The results suggested that the interaction between the ENS and inflammation plays a role in abdominal pain, with a pattern that mirrors what occurs in IBS [81,94]. We evaluated nerve fiber sprouting, that is the outgrowth of new fibers, in the colonic mucosa of asymptomatic and symptomatic patients with SUDD, compared to healthy subjects, in a region close to the diverticulum neck (diverticular region) and in a site far from diverticula. We stained sprouting fibers by using an antibody against growth-associated protein 43 (GAP43). Our results showed that the density of fibers was increased in both groups of patients with diverticula compared to controls in both regions analyzed. On the other side, sprouting fibers were significantly increased only in the diverticular region of patients with SUDD compared to controls and diverticulosis [93]. These results suggest that sprouting could be involved in symptom generation. Previously, we showed increased GAP43 positive fibers in the colonic mucosa of patients with IBS and increased levels of NGF which could be a key mediator of sprouting [100]. In addition, neuroplastic changes are involved in pain development and persistence [101]. Moreover, the inhibition of GAP43 induces the inhibition of neuropathic pain in animals, showing the role of GAP43 in visceral hypersensitivity [102].

Interestingly, we found that macrophages close to sprouting fibers were increased in the diverticular region of SUDD patients compared to healthy subjects, supporting a role for neuro-immune interactions in the pathophysiology of SUDD [93].

### 4.4. Muscular Layer and Neuro-Muscular Interactions

The longitudinal musculature of the colon, unlike in the esophagus, stomach, and small intestine, does not form a continuous layer, but rather consists of three parallel muscle bands called taeniae coli. The presence of these structures appears to predispose to the development of diverticula in the colonic parts devoid of these structures [103].

Studies conducted on the full-thickness colon of patients with diverticulosis revealed changes in molecules involved in smooth muscle contractility [104], increased connective tissue in the longitudinal muscle layer, reduced amount of myofilaments, and down-regulation of smooth muscle myosin heavy chain [105].

From a histopathological point of view, DD is characterized by a thickening of both circular and longitudinal muscle layers which seems to be due to hypertrophy of myocytes and increased deposition of connective tissue [105,106]. Despite this thickening, it has been seen that the smooth muscle myosin heavy chain, an essential component of the myofilament, is decreased in both gene and protein expression in patients with DD [105]. This finding could indicate a phenotypic shift from smooth muscle cells to fibroblast-like cells, resulting in decreased functioning myofilaments and altered functional integrity of the muscular apparatus in DD [105].

In vitro experiments conducted on intestinal smooth muscle samples of patients with DD, have revealed altered responses after exposure to excitatory and inhibitory or agonist neurotransmitters. Most studies have found impaired relaxation capacity of diverticular specimens due to an impaired nitrergic neurotransmitter system [96,107], except for one study reporting increased nitric oxide-mediated responses [108]. The alteration may also involve the receptors of neurotransmitters implicated in mediating intestinal motility, such as an up-regulation of muscarinic receptor 3 (M3) and down-regulation of serotonin receptor 4 (5HT-4R) [97,109].

The analysis of colonic circular smooth muscle of SUDD patients undergoing surgery revealed an increased sensitivity in vitro to exogenous acetylcholine (ACh) [97,110] and tachykinergic agonists [111]. These findings, taken in conjunction with a decrease in smooth muscle choline acetyltransferase activity and upregulation of postsynaptic muscarinic M3 receptors, raised the hypothesis that a type of hypersensitivity to cholinergic denervation occurs in SUDD [97], a mechanism that also occurs following damage to skeletal muscle motor innervation. Finally, the lowest number of glial cells observed in these patients could be another reason for the altered intestinal motility [112].

## 5. Future Perspectives

Understanding the molecular mechanisms involved in the development of diverticular disease and in symptom generation is crucial to advancing personalized approaches to the treatment and to identify biomarkers. State of the art technologies, the inclusion of a large cohort of patients, as well as of a control cohort, and the translational approach will be crucial to define the role of genetic, environmental, and micro-environmental factors in DD and will ultimately improve the management of the disease, and spread the development of novel therapeutic strategies and preventive approaches.

## 6. Conclusions

**Figure 1:**
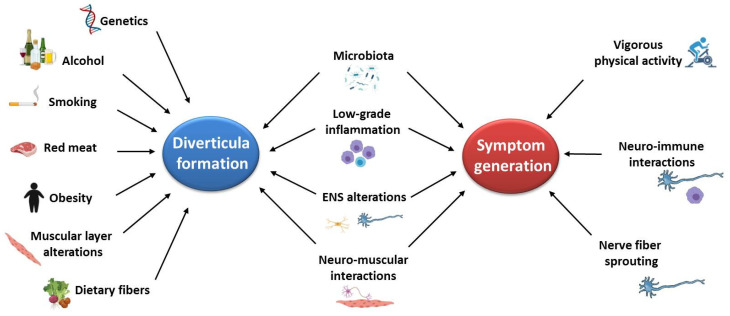
Factors involved in diverticula formation and/or in symptom generation. On the basis of the available data, the figure shows which factors are likely involved in diverticula formation (i.e., in the pathophysiology of diverticulosis and SUDD) and/or in symptom generation (i.e., in the pathophysiology of SUDD).

Despite tremendous advancement in our knowledge concerning the epidemiological, genetic, and pathophysiological aspects of DD, a common view on the pathogenesis of diverticula formation and the evolution towards symptoms and complications remain to be clarified [7,113,114]. Based on the available evidence, some factors are likely involved in diverticula formation, others in symptom generation, and others in both (Figure 1).

Genetic factors are likely key elements, involved in the pathophysiology of DD and, in the future, could open a better understanding of DD and open new avenues in the treatment of this condition, however, GWAS studies are still too limited. Although the role of the environment in DD is undisputed, there are still conflicting data on dietary fibers, one of the first factors studied in DD pathophysiology.

**Figure 2:**
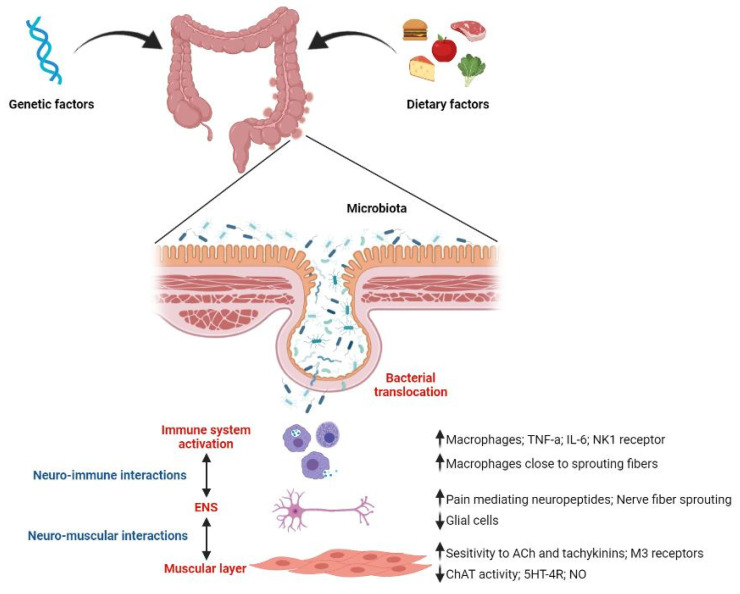
Representative figure of pathophysiological mechanisms involved in SUDD. ENS, enteric nervous system; Ach, acetylcholine; ChAT, choline acetyltransferase; NO, nitric oxide; TNF-a, tumor necrosis factor alpha; IL-6, interleukin-6; NK1, neurokinin1; 5HT-4R, 5idrossitriptamina-4 receptor; ↓, decrease; ↑, increase; ↕, bidirectional interaction.

The presence of diverticula can predispose to the weakness of the epithelial barrier that can likely become a point of entrance for the bacteria and other luminal antigens into the lamina propria. This may induce hyperactivation of immune cells with subsequent effects on the ENS and muscular cells which could explain symptom generation in SUDD (Figure 2).

This hypothesis remains to be demonstrated by experimental evidence, although some data are supporting it. Gut microbiota imbalance, neuro-immune, and neuro-muscular alterations have been reported in DD, although technical differences among studies and small sample size are still a limit to draw conclusions. Unfortunately, very limiting data are available on epithelial barrier alterations to confirm or reject the above hypothesis. Future studies, in larger cohorts, are desirable to better understand the pathophysiological mechanisms underlying diverticula formation as well as symptom generation, in order to develop a tailored therapy for the different clinical settings of DD and to prevent complications.

## Figures and Tables

**Table 1:** Main studies assessing genetic variants in DD.

Authors	Subjects (n)	Genome Profiling Method	Outcomes
Reichert et al., 2018 [23]	Diverticulosis (422) HC (285)	TaqMan assays	Positive association between the *COL3A1* variant and the risk of developing diverticulosis.
Connelly et al., 2014 [24]	Diverticulosis (21) HC (21)	TaqMan assay	Positive association of the single nucleotide polymorphism rs7848647 in the *TNFSF15* gene with diverticulitis requiring surgical intervention.
Sigurdsson et al., 2017 [25]	DD (11′396) HC (248′971)	GWAS	Variants in introns of the *ARHGAP15*, *COLQ* and *FAM155A* associate with diverticular disease or diverticulitis.
Coble et al., 2017 [26]	Diverticulitis (153)	Exome sequencing	*LAMB4* variants were identified in patients with diverticulitis.
Nehring et al., 2021 [27]	Diverticulosis (100) HC (120)	PCR–restriction fragments	The SNP rs4898 in *TMP1* gene correlates with an increased risk of diverticulosis.
Reichert et al., 2020 [29]	Diverticulosis (856) HC (479) Diverticulitis (198)	Taqman assays	Association of *ARHGAP15* and *COLQ* variants with uncomplicated DD and *FAM155* mutation with diverticulitis, but not with diverticulosis.
Maguire et al., 2018 [37]	DD (27,444) HC (382,284)	GWAS	DD is associated with 42 loci localized in genes implicated in immunity (*ARHGAP15, FADD, HLX*), cell adhesion (*BMPR1B, CLSTN2, COL6A1, CRISPLD2, EFEMP1, ELN, ENPP2, HAS2, IGSF10, LIMK1, LRRC17, NOV, PCSK5, S100A11, SHFM1, TCHH*), membrane transport and signaling (*ANO1, CACNB2, CALCA, CALCB, CHRNB1, COLQ, CUTC, S100A10, SLC25A28, SLC35F3,* SPINT2), and intestinal motility (*ANO1, CHRNB1, COLQ, PPP1R14A*).
Schafmayer et al., 2019 [38]	DD (31,964) HC (419,135)	GWAS	Discovered 48 risk loci close genes (*ARHGAP15, FAM155A, COLQ, GPR158, ABO, ANO1/FADS, ELN, BMPR1B, SLC35F3, SEM1/SHFM1, CTAGE1, NOV, CALCB, S100A10, DISP2, CACNB2, HLX, EDEM1, EFEMP1, LYPLAL1-AS1, SLC25A28, CWC27, SLC4A1, AC103796.1, CRISPLD2, WDR70, HAS2, PCSK5, NT5C1B, TRPS1*) impaired in intestinal neuromuscular function, mesenteric vascular, smooth muscle, and connective fiber support associate with DD.
Choe et al., 2019 [39]	Diverticulosis (893) HC (1075)	GWAS	Identified 9 SNPs located in *WNT4*, *RHOU*, and *OAS1/3* genes.

HC, healthy controls; GWAS, genome-wide association studies; COL3A1, Collagen Type III Alpha 1 Chain; TNFSF15, Tumor Necrosis Factor Superfamily Member 15; ARHGAP15, Rho GTPase Activating Protein 15; COLQ, Collagen Like Tail Subunit Of Asymmetric Acetylcholinesterase; FAM155A, Family with sequence similarity 155 member A; LAMB4, Laminin Subunit Beta 4; TMP1, Tropomyosin 1 alpha; FADD, Fas Associated Via Death Domain; HLX, H2.0 Like Homeobox; BMPR1B, Bone Morphogenetic Protein Receptor Type 1B; CLSTN2, Calsyntenin 2; COL6A1, Collagen Type VI Alpha 1 Chain; CRISPLD2, Cysteine Rich Secretory Protein LCCL Domain Containing 2; EFEMP1, EGF Containing Fibulin Extracellular Matrix Protein 1; ELN, Elastin; ENPP2, Ectonucleotide Pyrophosphatase/Phosphodiesterase 2; HAS2, Hyaluronan Synthase 2; IGSF10, Immunoglobulin Superfamily Member 10; LIMK1, LIM Domain Kinase 1; LRRC17, Leucine Rich Repeat Containing 17; NOV, Cellular Communication Network Factor 3; PCSK5, Proprotein Convertase Subtilisin/Kexin Type 5; S100A11, S100 Calcium Binding Protein A11; SHFM1, SEM1 26S proteasome subunit; TCHH, Trichohyalin; ANO1, anoctamin 1; CACNB2, Calcium Voltage-Gated Channel Auxiliary Subunit Beta 2; CALCA, Calcitonin Related Polypeptide Alpha; CALCB, Calcitonin Related Polypeptide Beta; CHRNB1, Cholinergic Receptor Nicotinic Beta 1 Subunit; COLQ, Collagen Like Tail Subunit Of Asymmetric Acetylcholinesterase; CUTC, CutC Copper Transporter; S100A10, S100 Calcium Binding Protein A10; SLC25A28, Solute Carrier Family 25 Member 28; SLC35F3, Solute Carrier Family 35 Member F3; SPINT2, Serine Peptidase Inhibitor, Kunitz Type 2; CHRNB1, Cholinergic Receptor Nicotinic Beta 1 Subunit; PPP1R14A, Protein Phosphatase 1 Regulatory Inhibitor Subunit 14A; GPR158, G Protein-Coupled Receptor 158; ABO, Alpha 1-3-N-Acetylgalactosaminyltransferase And Alpha 1-3-Galactosyltransferase; CTAGE1, cutaneous T-cell lymphoma-associated antigen 1; DISP2, Dispatched RND Transporter Family Member 2; LYPLAL1-AS1, LYPLAL1 Antisense RNA 1; CWC27, CWC27 Spliceosome Associated Cyclophilin; AC103796.1, brain derived neurotrophic factor; WDR70, WD Repeat Domain 70; NT5C1B, 5’-Nucleotidase, Cytosolic IB; TRPS1, Transcriptional Repressor GATA Binding 1; WNT4, Wnt Family Member 4; RHOU, Ras Homolog Family Member U; OAS1/3, 2’-5’-Oligoadenylate Synthetase 1.

**Table 2:** Main studies assessing fiber role in DD.

Author	Subjects (n)	Diet	Outcomes
Burkitt DP et al., 1972 [44]	General population (1200)	Low-fibers Western diet vs. high-fiber diet	Low-fiber Western diet produces smaller stool volumes and longer transit time, with the consequent increase of intraluminal pressure predisposing to diverticular herniation.
Aldoori et al., 1998 [45] Aldoori et al., 1994 [52]	General population (male) (43,881)	High soluble fiber assumption	Decrease risk to develop DD.
Crowe et al., 2011 [46]	General population (47,033)	Vegetarian diet and dietary fiber assumption	Vegetarians had a risk reduction of 30% to develop DD compared to subjects consuming meat. Individuals consuming more than 25 g of fibers/day had a decrease of 40% in the hospitalization risk compared to subjects consuming less than 14 g/day.
Peery et al., 2012 [53]	General population (2104)	High-fiber diet	High fiber diet associates with a higher prevalence of diverticulosis.
Peery et al., 2013 [47]	Diverticulosis and controls (2108)	Low-fiber diet	No association between constipation and the risk of diverticulosis.
Aune et al., 2020 [48]	General population (876,111; 19,282 cases and 856,829 participants)	Free diet	Subjects consuming a high fiber diet (30 g per day) had a reduction of 41% of the risk to develop DD, compared to subjects with a low fiber diet.

## Data Availability

The data presented in this study are openly available in Medline and Embase.

## Abbreviations

Diverticular disease, DD; symptomatic uncomplicated diverticular disease, SUDD; Irritable bowel syndrome, IBS; inflammatory bowel disease, IBD; segmental colitis associated with diverticulosis, SCAD; single nucleotide polymorphisms, SNPs; genome-wide association studies, GWAS; Gastroenterology Association, AGA; short-chain fatty acids, SCFAs; C-reactive protein, CRP; neurokinin 1 receptor, NK1; glial cell-derived neurotrophic factor, GDNF; Rearranged during transfection, RET; acetylcholine, ACh.
